# Nouveau Traité De L’Accouchement Manuel, Ou Contre Nature, Réduit à Sa Plus Grande Simplicité Par L’Analogie Des Positions Diagonales De Toutes Les Régions Du Tronc Fœtal Avec Les Positions De L’Occiput

**Published:** 1841-02-20

**Authors:** 


### BIBLIOGRAPHICAL NOTICE.

*Nouveau Traité de l'Accouchement Manuel, ou contre Nature, réduit à sa plus grande simplicité par l'analogie des positions diagonales de toutes les règions du tronc fœtal avec les positions de l'occiput.* Par J. M. Le Monnier, Docteur en Chirurgie, Professeur particulier d'Accouchements, des Maladies des Femmes et des Enfants, ex-Professeur de Anatomie, de Physiologie et de Médecine Opératoire, a Rennes, (Ille-et-Vilaine.)

WE have just received a copy of this work, which was issued in numbers, and accompanied by folio plates. These exhibit every presentation requiring manual delivery. The execution of the plates is not, however, worthy of the text. The latter is the production of a scientific mind—the former the effort of an artist who has certainly never made anatomy his special study. Professor Le Monnier, whose recent demise we are called upon to deplore, was for a long period a distinguished teacher and practitioner of midwifery in France. At Rennes, where he resided during the latter years of his life, he stood unequalled, and his loss will be deeply felt. The work before us bears ample testimony to the originality of his mind, and to the unceasing attention which he bestowed upon that branch of his profession which he had adopted as a speciality. To the student of midwifery, his work will be a most valuable guide.

